# Reasons for implementation success despite health system constraints: qualitative insights on ‘what worked’ for cotrimoxazole preventive therapy

**DOI:** 10.1186/s12913-024-10631-x

**Published:** 2024-03-27

**Authors:** Pia Müller, Edna Mabasso, Luís Velez Lapão, Mohsin Sidat

**Affiliations:** 1 Instituto de Higiene e Medicina Tropical, Universidade Nova de Lisboa, Lisbon, Portugal; 2 Faculdade de Medicina, Universidade Eduardo Mondlane, Maputo, Mozambique; 3 UNIDEMI, Department of Mechanical and Industrial Engineering, NOVA School of Science and Technology, Universidade NOVA de Lisboa, Caparica, Portugal; 4 Laboratório Associado de Sistemas Inteligentes, LASI, Guimarães, 4800-058 Portugal; 5 WHO Collaborating Center on Health Workforce Policy and Planning, Instituto de Higiene e Medicina Tropical, Universidade NOVA de Lisboa, Lisbon, Portugal

**Keywords:** HIV, Preventive care, Kit system, Supply chain, Service delivery, Integrated care, Differentiated care, Pharmaceutical production, Low-income country

## Abstract

**Background:**

Although Cotrimoxazole preventive therapy (CPT) has shown to be highly efficacious in reducing morbidity and mortality among people living with Human immunodeficiency virus (HIV) under ‘ideal world’ study conditions, operational challenges are limiting its effectiveness when implementing in countries most affected by the HIV epidemic. The fact that Mozambican authorities reported high coverage of CPT among patients with HIV, has led to this qualitative case study aimed at exploring possible factors responsible for the successful implementation of CPT in the Province of Maputo.

**Methods:**

Between February and April 2019, we individually interviewed nine governmental stakeholders, including the person responsible for the HIV Program, the person responsible for the TB Program and the person responsible for Pharmaceutical management at three administrative levels (central, provincial and district level). Interviews were recorded, transcribed, and analysed thematically using MAXQDA Analytics Pro. Findings were translated from Portuguese into English.

**Results:**

Five themes iteratively emerged: (a) Role of governance & leadership, (b) Pharmaceutical strategies, (c) Service delivery modifications, (d) Health care provider factors, and (e) Patients’ perspectives.

Interviews revealed that continuous supply of cotrimoxazole (CTZ) had been facilitated through multiple-source procurement and a push-pull strategy. One part of CTZ arrived in kits that were imported from overseas and distributed to public health facilities based on their number of outpatient consultations (push strategy). Another part of CTZ was locally produced and distributed as per health facility demand (pull strategy). Strong district level accountability also contributed to the public availability of CTZ. Interviewees praised models of differentiated care, the integrated HIV service delivery and drug delivery strategies for reducing long queues at the health facility, better accommodating patients’ needs and reducing their financial and organisational burden.

**Conclusions:**

This study presents aspects that governmental experts believed to be key for the implementation of CPT in the Province of Maputo, Mozambique. Enhancing the implementation outcomes – drug availability and feasibility of the health facility-based service delivery – seemed crucial for the implementation progress. Reasons for the remarkable patient acceptability of CPT in our study setting should be further investigated.

**Supplementary Information:**

The online version contains supplementary material available at 10.1186/s12913-024-10631-x.

## Background

### The role of CPT historically

In the early 1980s, the Human immunodeficiency virus (HIV) was recognised as a worldwide epidemic. However, in most parts of sub-Saharan Africa, this epidemic was acknowledged later at a time when HIV prevalence and mortality attributed to HIV infection or the acquired immune deficiency syndrome (AIDS) were already high in the general population. For two decades, there was little hope for those diagnosed with HIV infection, facing progressive immunosuppression and certain death within few years. Although the first triple antiretroviral therapy (ART) was introduced in the mid-1990s, due to its’ prohibitively high cost at that time, this combination therapy was out-of-reach for African governments [1]. In 2000, cotrimoxazole (CTZ) preventive therapy (CPT) became the first evidence-based ‘conservative medicine’ promising to reduce morbidity and mortality among people living with HIV (PLHIV) within reach for low and middle income countries (LMICs) [2–4]. Due to its intrinsic activity against a broad spectrum of bacterial, fungal and protozoal agents, the inexpensive drug combination (trimethoprim/ sulfamethoxazole) offered an opportunity to prevent common coinfections in people with advanced HIV disease that frequently cause death [5]. However, emerging evidence from LMICs demonstrated clinical benefits of CPT beyond substantial reductions in these opportunistic infections, even after immune reconstitution on ART [6, 7] and despite high levels of microbial resistance [8, 9]. These clinical benefits include reductions in malaria and severe bacterial infections [5]. As a result, CPT has not been completely replaced by ART during its large scale-up in Africa since the mid-2000s. At least until ART-induced immune recovery or viral suppression is achieved, CPT is still strongly recommended for PLHIV today [5].

### Implementation challenges

Despite the solid evidence demonstrating the efficacy and cost-effectiveness of CPT, research translation into practice has been slow [5, 10]. In their comparative analysis, Hutchinson et al. [10] explored factors influential in the research-to-policy process for CPT in Uganda, Malawi and Zambia. Their study revealed that context (including the influence of donor agencies), networks and links between individuals involved in research and policy-making, and the nature of the evidence available had an influence on national policy creation [10, 11]. The governments in all three countries studied had considered the evidence behind the 2000 provisional recommendations [4] on the use of CPT as insufficient to justify implementation. Consequently, national policy was delayed in Uganda, Malawi and Zambia until 2003, 2005, and 2006 respectively, the latter partly due to funding issues [10]. Beyond delays in the research-to-policy process, major challenges to translate CPT policy into clinical practice, have been reported. In our recently published systematic review, we explored barriers to implementing CPT across countries with a high burden of Tuberculosis (TB) and HIV and generated explanatory knowledge of why implementation has been so challenging [12]. We identified eleven studies from six countries reporting barriers to CPT, all located in sub-Saharan Africa (Malawi, South Africa, Tanzania, Uganda, Zimbabwe and Lesotho) [13–23]. These LMICs are historically dependent on international funding [24] that has been mainly channelled into vertical health Programs (e.g. HIV/AIDS-, TB-, child health Program), typically associated with vertical governance and fragmented service delivery [18, 24]. After all, CTZ is an inexpensive generic drug, but governments lacked funds, leadership and coordination to ensure its collaborative implementation [14, 21]. Consequently, CPT was frequently offered at HIV/AIDS services only, requiring patients (e.g. pregnant women, children, people seeking TB services) referral between sub-speciality services or clinics [13–15, 21]. This operational fragmentation often impeded the quality and efficiency of service delivery and added to patients’ transport costs and lengthy time spent in queues [16, 21, 23]. Task shifting has been an important strategy to address the severe health workforce shortages in the sub-Saharan Africa region [25], where low and mid-level healthcare providers usually deliver or dispense preventive therapy [14, 15, 20]. However, these healthcare providers lacked knowledge or training on CPT [14, 15, 20, 21]. Another critical obstacle to implementing the life-saving intervention was that health facilities and pharmacies frequently ran out of CTZ [13, 15, 16, 18, 21, 23]. Health facility level reasons for stock-outs of CTZ included an increasing number of patients with HIV/AIDS [21], challenges with forecasting drug demand [15, 21], late submission of requisitions [21], ordering inadequate quantities [15] and a lack of funds to purchase medicines [16]. Poor recording of CPT on patient files has been reported, undermining the facility-based drug demand [14, 17]. The disproportional provider-patient ratio [25] and weak information systems that lacked integration and relied on both paper-based and electronic files provided an explanation for inconsistent recording practices [14, 15, 17, 18, 21]. Scarcity of CTZ at the medical stores’ department or government supplier side [16], ineffective supply chain management [15, 18] a lack of support from policymakers, Program managers or district health departments has also been reported [15, 18]. To sum up, we identified barriers to the implementation of CPT across all building blocks of the health system rather than major intervention-specific issues [12]. Considering fragile public health systems, the implementation of CPT is undoubtedly more challenging in LMIC in sub-Saharan Africa than in wealthier parts of the world.

### Mozambique’s positive experience with implementing CPT

Mozambique is a low-income country with an estimated 13.2% of the adult population living with HIV and over 35,000 deaths associated with HIV/AIDS, in 2021 [26]. Almost half (46.1%) of the country’s 31 million inhabitants are trapped in chronic poverty, and the national HIV/AIDS response strongly depends on foreign aid. More than 97% of the HIV/ AIDS Program funds were contributed by international donors (principally the US President’s Emergency Plan for AIDS Relief (PEPFAR) and the Global Fund to fight AIDS, TB and Malaria (GFATM)) [16, 17]. Nevertheless, over the past two decades, the country achieved large gains in life expectancy, maternal health, and in improving access to health, considering the obstacles the country has faced since its independence from Portugal 1975, including a 16 year civil war that ended in 1992, the HIV/AIDS epidemic and recurrent natural catastrophes [19, 21]. Despite existing health system challenges, the country reported considerable success with implementing CPT [27].

## Methods

### Rationale

During a scientific meeting held by MS at the Eduardo Mondlane University in Maputo 2017, local doctors, researchers and governmental stakeholders argued that the implementation of CPT had been achieved with great success in ‘their Province’. Mozambican Ministry of Health authorities confirmed this statement, noting that national level Program targets had been achieved over consecutive years, so that performance monitoring of CPT as a routine indicator was discontinued, in 2018 ([27]; p. 51). In this study we aimed to explore possible factors responsible for the successful implementation of CPT (with special attention to facilitators that may have helped to overcome barriers frequently identified in other high TB/HIV burden countries) [12]. Another aim of this study (to be published elsewhere) was to explore stakeholders’ thoughts about why isoniazid preventive therapy has been more challenging to implement in the same setting when comparing to CPT.

### Theory and methodological orientation

Qualitative research generates rich information and is very valuable for studying dynamic contextual factors affecting implementation. In contrast to traditional qualitative research that is influenced by a clear cut constructivist paradigm, qualitative methods within implementation science tend to be more positivist and deductive in nature [28]. Our qualitative study design emerged from a constructivist and postpositivist worldview and includes elements of phenomenology and participatory action research with interviews as primary method for data collection, followed by a focus group discussion [29]. When designing the study, we determined that interviewing governmental stakeholders (who possess extensive knowledge on policy changes, organisational and structural alterations within the health system), would be a meaningful approach to generate understanding of which factors may have contributed to the successful implementation of CPT in the Province of Maputo. To complement the perspectives gained from individual interviews, a focus group discussion was carried out to promote critical reflection and discussion about the lessons learnt and to verify eventual saturation of information obtained during interviews.

### Participants

Nine governmental stakeholders including the person responsible for the HIV Program 1 the person responsible for the TB Program and the person responsible for pharmaceutical management at three administrative levels (central, provincial and district) were purposively selected for interviews. We selected the District of Matola that is one of eight districts located in the Province of Maputo for interviews with each of the three respective stakeholders. Matola district was selected (i) because of the similar HIV profile in the district Matola and Maputo Province (with about 23% of adults aged 15-49 years living with HIV, respectively) [26, 30], (ii) for the reason that more than one quarter of the province’s population resides in this district, and (iii) because the district is comprised of urban, sub-urban and rural areas, reflecting contextual diversity [31].

### Interview procedure

Interviews were scheduled between February and April 2019 and carried out individually, face-to-face at each expert’s office located at the Ministry of Health (MoH), Provincial and District Department of Health (PDH, DDH). Two female researchers trained in qualitative research methods, without any personal connection to the interviewees conducted the interviews collaboratively in the local language (Portuguese) [32]. First, PM a non-native speaking doctoral researcher specialised in infectious diseases control presented the rationale behind the study, its objectives, ethical considerations, and answered questions regarding the study. Then, EM a native speaking local medical doctor obtained written consent from the governmental expert to participate, and requested authorisation to audio-record the interview. A pilot-tested semi-structured interview guide was applied by EM who was as non-directive as possible in her speech and encouraged the interviewee to give a full description of their thoughts to each question, including their experiences and opinions. The interview guide (see Additional file 1) included an initial list of open-ended questions with flexible order. Depending on the interviewee’s answers, spontaneous probes or follow-up questions were generated by both interviewers to seek for further detail. Interviews were completed within 60 min.

### Data analysis

Interview recordings were transcribed (by EM) and analysed by both interviewers using MAXQDA Analytics Pro. A narrative approach using an emergent strategy was taken for thematic analysis of text data. After carefully reading all transcripts, both data analysts (PM and EM) individually analysed each transcript line by line, iteratively segmenting sentences or paragraphs into codes and sub-codes that represented central ideas. Through several rounds of coding, both researchers individually defined, cross-checked, discussed, and refined the code system accordingly. Disagreement was resolved through discussion. The main themes that emerged in the final round of coding were presented with description, supported by direct quotations from interview transcripts [33]. Main themes, description and quotations were translated into English by PM, cross-checked and discussed with EM. We then applied thematic network analysis, a well-established and highly sensitive technique for systematisation and presentation of qualitative analysis, that has proven particularly useful when numerous themes (≥ 4) emerge from textual data [34]. We grouped identified themes (basic themes) together as categories (organising themes) and presented them according to the underlying story they are telling. We reported our findings, according to the COREQ (Consolidated criteria for Reporting Qualitative research) checklist (see Additional file 2) [35].

### Content verification (qualitative validity)

Findings obtained from the audio transcripts were shared with each interviewee with the request to verify portions of the data, and providing an opportunity to them to modify, delete or add detail to the findings. In April 2019, the interview participants and additional stakeholders involved in the policy implementation were invited once again this time to participate in a two-hour long focus group discussion held by PM and EM with the aim to confirm the accuracy of qualitative findings and to facilitate a dialogue between stakeholders. First, the main themes that emerged during data analysis were presented to the focus group, followed by discussion about whether the participants felt that the findings presented were accurate and representative. Focus group discussion was audio-recorded, transcribed and analysed for complementary information, and data saturation (assessing if new codes emerged from focus group data) [29].

## Results

### Interviews

All nine governmental stakeholders who were invited for interviews participated; none dropped out or refused. Table 1 provides a list of the fifteen themes that emerged as facilitating factors for the implementation of CPT.

**Table 1:** Identified themes organised by category, and expert that contributed information to each theme

Themes, ordered by category	**Experts’ hierarchical level and area of responsibility**								
	Central level (MoH/CMAM)			Provincial level (PDH)			District level (DDH)		
	HIV	TB	Pharmaceuticals	HIV	TB	Pharmaceuticals	HIV	TB	Pharmaceuticals
**Governance contributions**									
Importance of policy, protocol and guideline	X	X	X		X		X		
Collaborative elaboration of documents and implementation strategies	X	X	X			X			
Continuous improvement	X	X	X	X					
**Pharmaceutical strategies**									
Prioritisation of cotrimoxazole	X		X			X			
Training on logistics, pharmaceutical management & operating procedures		X				X			
Providing CTZ through two distinct distribution channels: ‘kit system’ & ‘standard path delivery’		X	X		X	X			X
Local production of cotrimoxazole			X			X			X
District level strategies			X			X	X	X	X
**Service delivery improvements**									
Patient-centered care				X	X	X	X	X	X
Integration of care	X	X			X		X	X	X
Differentiated care				X	X		X		X
Drug delivery strategies				X	X	X	X	X	X
**Health care providers' factors**									
Stock of health professionals				X					
Training & health providers' acceptability to prescribe CPT	X	X					X	X	
**Patients' perspective**									
Patients’ strong preference for cotrimoxazole	X			X			X	X	

*Abbreviations*: *MoH* Ministry of health, *CMAM* Central institution for Medicines and Medical Supply, *PDH* Provincial Department of Health, *DDH* District Department of Health

### Role of governance & leadership

#### Importance of policy, protocol and guideline

Central level experts highlighted the importance of policy, protocol and clinical guidelines, which were described as clear and straightforward for CPT. Policy updates were circulated, discussed and displayed at all levels. It was noted that policy never included the legal requirement for doctors to prescribe CTZ, permitting nurses and other lower-level cadre to prescribe or deliver CPT since the beginning of implementation.

*We [Ministry of Health] established a clear guideline, based on WHO recommendations. [For patients with] CD4 cells below 350, we are offering [CPT]. It is crystal clear." (HIV Program Director, MoH)*

#### Collaborative elaboration of documents and implementation strategies

Interviewees pointed out that essential documents and implementation strategies regarding CPT were collaboratively elaborated during regular technical working group 2 meetings at the central level. Typically, HIV and TB Program Directors, an expert responsible for the control of opportunistic infections, and pharmaceutical experts collaboratively define strategies.

*“We [technical group members] analyse the WHO recommendation and see [how this fits into our reality]. …We understood the kind of support needed from CMAM and decided together about the best timing to start implementation.” (Pharmaceuticals Director CMAM)*

#### Continuous improvement

Interviewees suggested that ongoing efforts have been made to reach the current level of implementation, referring to the continuous availability of CTZ at the frontlines, and the high coverage of CPT among PLHIV. Monitoring and evaluation activities, supervision visits and weekly health facility meetings have been mentioned as strategies that have helped to identify problems in a specific district or health facility, and rapidly take adequate action.

*“We discuss the provision of CTZ during our weekly health facility (ART) meetings, which helped a lot because that way, the facility itself managed to respond adequately according to its own reality. Cotrimoxazole perhaps has been a challenge in the past. But now, I believe that we have managed to overcome the problems.” (HIV Program Manager, PDH)*

Due to the successful implementation of CPT, at present, there is still no routine indicator dedicated to monitoring progress regarding the implementation of CPT, but CPT may still happen to be subject of an evaluation, supervision visit, or topic during the weekly health facility meeting, experts explained, when probed.

*“What is there to be improved about CPT, an intervention with 98% coverage? There is not such a thing as a public health intervention that achieves 100%." (HIV Program Director, MoH); "Somehow we did several things right when it comes to the implementation of CTZ, shown in an excellent [stock availability and] coverage.” (Pharmaceuticals Director CMAM)*

### Pharmaceutical strategies

Ensuring the availability of CTZ was perceived as key for the successful implementation.

*“What really helped to improve the implementation of CTZ was to ensure the availability of the medication.” (TB Program Director, MoH)*

#### Prioritisation of cotrimoxazole

CTZ has been mentioned as priority of the HIV program and of CMAM, with a strategic focus on ensuring funding for and the availability of CTZ.

“Cotrimoxazole has always been a priority for us [HIV/AIDS Program] as a measure to prevent opportunistic infections, and as we are in a perennial endemic area for malaria, it is also important for malaria prevention.” … “The circle of CPT provision is never interrupted. And we know very well what constant stockouts means; it causes health providers not to prescribe even when stock is available [again], but we never had this problem with CPT.” (HIV Program Director, MoH); "We [government] have a list of what is extremely prioritised, for which funding has always been guaranteed. We buy 100% of what is needed, no matter how much we receive from our partners [donors]. We don’t allow any gap for this item." (Pharmaceuticals Director CMAM);

#### Providing CTZ through two distinct distribution channels

The pharmaceutical experts pointed out that CTZ was provided through two distinct channels, referring to the automated supply of kits and the health facility specific requisition of individual medicines. Experts reflected on key characteristics of both supply channels, namely ‘kit system’ and ‘standard path delivery’ also known locally as ‘via clássica’ (Table 2).

**Table 2:** Key characteristics of ‘kit system’ and ‘standard path delivery’ for the supply of CTZ

	**Kit system**	**Standard path delivery (‘via clássica’)**
Meaning	Kit: Box with pre-defined number of essential medicines and medical supply (altogether nearly 60 items)	Health facility specific requisition of any medicine/ medical supply included on the list of publicly funded medicines
Combination supply chain strategy	Basic stock via push system: Automated delivery of prepacked kits that include CTZ based on each facility's number of outpatient consultations	Top up of stock via pull system: Additional delivery of CTZ as per requisition (if the demand exceeds the quantities available at the health facility level)
Stakeholder(s) responsible for quantification	Technical group for quantification at the central level (comprised of CMAM, HIV Program, Data manager), and representatives of principal partners (i.e. Global Fund, PEPFAR, MSF)	Health facility pharmacy personnel
Quantification [method]	[Morbidity method] • Procurement: Calculating the theoretical quantity of CTZ for one year (in advance) considering: - CTZ as preventive treatment for PLHIV - CTZ as antibiotic treatment of bacterial infections among the general population • Automated delivery: supplying quantity of kits per health facility based on monthly mean number of outpatient consultations	[Consumption method] • Health facility-based requisition: electronic generation and submission of a monthly report (that includes all data needed for quantification) via one of two logistics information systems: SIMAM (central level platform used at high patient turnover facilities) or SIGLUS (newer platform used at lower patient turnover facilities) • Calculating the quantity of additional CTZ needed for the ongoing month, considering: - Total quantity of CTZ needed for the month (likely consumption based on the previous month's number of outpatient consultations registered through SESP (electronic information system for patient follow-up)) - Subtracting existing quantity of CTZ available at the health facility (stock inventory) - Multiplying by 2: to include security (buffer) stock
Planning	1. Selecting list of essential medicines and medical supply items for kits 2. Determining quantities for each item per one kit, which is designed for 1000 outpatient consultations 3. Inviting tenders to provide offers for kit supply contracts 4. Contracting tenders, based on best price and quality of test kit content and packaging	1. Inviting local pharmaceutical companies to provide their best price for large quantities of CTZ 2. Contracting local company to produce and deliver CTZ to central level warehouses on a monthly basis
Pharmaceutical procurement	Purchasing pre-packed kits from overseas	Purchasing of CTZ from local pharmaceutical company (STRIDES; April 2019)
Supply chain	Supply chain: Central warehouse ➔ provincial warehouse ➔ (district warehouse* ➔) health facility *District warehouse had been built in Matola, but was not yet operational. (Status May 2020)	
Delivery to health facility	Beginning of each month	Middle of each month (5th to 15th day of a month)

Several experts suggested that the automated *kit supply* is important for ensuring the availability of basic stock quantities of CTZ at all health facilities irrespective of requisition. However, one expert noted that it is difficult to accurately quantify the drug demand one year in advance, which is necessary due to the long procurement process for kits imported from overseas. For kits, the last supply step (from district to health facilities) was frequently covered by non-governmental organisations (NGO’s) or other health sector personnel, facilitated through the straightforward receipt of a sealed kit.

*“Our biggest constraint lays in the last supply step: from district to the health facilities, but… you wouldn’t really notice because CTZ is included in kits, which is an extreme advantage. That way it [CTZ] reaches health facilities even in peripheral areas. Anybody passing by those health facilities can deliver a kit… NGO’s pass by, they pass by from the district warehouse. Something [kit] that is already in a proper sealed box is easier to deliver than something [standard path delivery items] that needs a stock count [for all items upon delivery]. Anybody I know can drop off a kit, anybody with responsibility. For ‘the other’ [standard path delivery], it has to be one of us, somebody with expertise.” (Pharmaceuticals Director CMAM)*

*Standard path supply* allows monthly health facility specific requisition of additional quantities of essential medicines (e.g. CTZ), and more specialised drugs not included in kits. Due to frequent delays for ‘via clássica’ 3 supply, this channel includes a higher margin of buffer stock, supplying health facilities with twice the stock requested to account for potential future delays. Another advantage of CTZ provided ‘via clássica’ is that it is produced by a local pharmaceutical company located within the district (Matola).

*“Health facilities often do it right [requisition], but the waiting period for replacement is long, especially for health facilities in peripheral areas."…" It’s not because there are no medicines, but because they arrive late. Buffer stock helps when we are waiting for replacement. So if I order stock for one month, I will receive stock for one more month as buffer.” (Pharmaceutical manager, DDH);*

#### Local production of CTZ

Since the policy for national production of pharmaceuticals has been introduced in 2018, pharmaceutical companies began to emerge, facilitating immediate access to medicines without having to go through the long procurement process common for drugs imported from overseas, the central level pharmaceutical expert explained. He perceived local production of pharmaceuticals as important progress. Since STRIDES pharmaceuticals began to deliver CTZ monthly to central level warehouses, CMAM was able to reduce central level buffer stock, resulting in less capital tied-up in stock, and less warehouse space needed, he argued. However, time delays associated with standard path delivery, were preventing domestically produced CTZ from being the only source of CTZ circulated in Mozambique.

*“So this medication [CTZ] has much luck… The fact that we recently started to produce solid forms, I think is something evolutionary.” … “STRIDES could and would like to produce the total amount of CTZ [to cover national demand]. The problem is the distribution; if we withdraw CTZ from the kit system, we need to deliver *via* ‘classic path’, and this is not the most reliable path, especially in rural areas. That is why we maintain [it in] the other [kits].” (Pharmaceuticals Director CMAM).*

#### Training on logistics, pharmaceutical management and operating procedures

A Training package has been developed with an international partner NGO to address lacking expertise in logistics and pharmaceutical management across the health facilities in the Province of Maputo, one pharmaceutical expert reported. Pharmaceutical training is provided to new and existing employees. Pharmacy personnel learn about operating procedures and the application of the mathematical formula for the requisition of medicines.

*“The pharmacy technician is the one in charge of pharmaceutical management at the health facility. But when we didn’t have someone from that area, we had to allocate nurses or medical assistants to do the job. This was one of the biggest barriers in our Province, which we managed to overcome with training. We offer training on pharmaceutical management and operating procedures, aimed at ensuring the availability of medicines for all Programs. Training is not just for people that don’t know, for those new, or not from ‘the area’. People often know, but end up neglecting or forgetting. So we offer refresher training, also because things change over time. We have a database, with which we filter [identify] all health providers working at the facilities that have not yet received training.” (Pharmaceutical Manager, PDH)*

#### District level strategies

Each DDH team includes a pharmaceutical manager that plays an important role for ensuring the availability of medicines at the districts’ health facilities, experts reported. The pharmaceutical manager provides supportive supervision to health facility personnel in charge of pharmacy operations. This person was also described as contact person in case of a health facility based low run on stock, responsible for coordinating transfers of medicines and medical supply within the district.

*“He goes to health facilities that have difficulties in justifying and requesting stock and helps them. So health facilities started to have less problems. This is the job of the district manager: to ensure that they do the justifications right and in time, and to pressure the Province to provide stock.” (HIV Program Manager, DDH)*

Compensation strategies that help to ensure the continued availability of CTZ at the health facility were also identified. Experts described a step-wise stock-seeking approach, that begins with stock transfers within the health facility, followed by stock transfers between health facilities, prior to pressuring the provincial authorities to deliver. Experts pointed out that these transfers of stock were frequently delivered by NGOs, sometimes delivered by district managers themselves, or the ambulance vehicle.

*“One strategy to guarantee CTZ is based on the existing stock within the health facility. If there is no CTZ in the pharmacy, the maternal and child health unit has some. They allocate small amounts to another service for one week or so, until the next delivery, then they return it [CTZ].” “…We also have the support of other health facilities. We have a [WhatsApp] group, and there, everyone [health facility personnel] puts their difficulties, shortages, or concerns to share with the entire district, promptly report stock issues internally first. We check, if they [other health facilities] have any. If not, we request stock from the provincial warehouse. We coordinate with our partner.” (Pharmaceutical Manager, DDH)*

### Service delivery improvements

Several service delivery modifications have been described by the experts interviewed as innovative strategies that supported the implementation of CPT.

#### Patient-centred care

Experts described service delivery adjustments carried out to better address patients’ needs, such as the extension of health facilities’ opening hours. To address HIV stigma, both psychosocial support and health lectures provided in the health facility waiting area, have been introduced to raise the health literacy of patients and their community. Because patients often fear to disclose their HIV status, they have the right to freely choose a health facility for their HIV care that may be close or distant to their home.

*“Some health facilities are closing late. …and some pharmacies open early at 6am. So patients can collect their medicines before the other doors [health services] open, before it gets really busy” (TB Manager, DDS)*

*“Stigma and fear of discrimination is a fact and problem we are still dealing with. A few things [we did] to improve this issue was giving health lectures, providing treatments [ART and CTZ] during the consultation, and giving more privacy to the patient.” (TB Manager, DDS); “We explain that if patients live far [choose a distant health facility for their HIV care] they must consider the time it takes to get there. We recommend patients to take into account that the location [of the health facility] can bring benefits or harms for their health. If patients choose the health facility nearby, this will also improve the indicators for CPT.” (Pharmaceutical Manager, DDS)*

Several experts suggested that communicating drug information to patients has been an important strategy for the implementation of CPT, arguing that key messages support patients to make informed decisions, and actively engage them in their own long term health.

*During our morning health lectures [in the health facilities’ outside waiting area], we talk about effects in addition to those desired effects. We explain that in case anybody has any sign [potential side effect] he must return [to the health facility]. We use those lectures to explain how to take the medication, side effects that may appear, noting those effects that require suspending therapy.” (TB Manager, DDH)*

#### Integration of care

Integration of HIV/AIDS care into primary care sub-speciality services has been described as another facilitating factor for the preventive therapy. Experts most frequently referred to the provision of CPT (and ART) at TB sites, but also referred to the integration of HIV care into chronic care, and maternal and child health services (MCH).

*“Mozambique in general is implementing the ‘one-stop shop’ strategy, where patients with HIV receive concomitant attention for other diseases. CPT and ART are provided at the same time within the TB sector. This helps the patient, because he does not have to stay in another queue, and we have it [‘one-stop shop’] in various sectors [TB, MCH, chronic care].” (TB Manager, PDH)*

One expert also acknowledged efforts made to integrate traditional medicine into the health facility based provision of ‘western medicine’. Besides enabling traditional healers to recognise TB symptoms among people that seek their healing approach, the district strategy aims to encourage traditional healers to advise their patients to concomitantly seek advice from a health care professional.

*“Instead of going to the health facility, people often go to a traditional healer. …It is our responsibility (DDH) to train [selected] traditional healers [in each region] as activists. Some already have a bit of knowledge, at least they know the principal symptoms of TB. When they receive patients [with TB symptoms] they send them to the health facility. There are referral guidelines. And because we understand that patients that go to these healers, believe in this type of medicine, we send them back there [to the traditional healer] as well. …We have a committee at the health facilities that involves religious leaders, community leaders and traditional healers, who periodically meet [including DDH representatives] to see how the project is developing, if there are any problems…” (TB Manager, DDS).*

#### Differentiated care

Most experts suggested that the recent implementation of differentiated HIV service delivery has facilitated the implementation of CPT. Differentiated care applies to stable patients with a controlled viral load and good adherence to ART. These patients may receive six-monthly consultation and a higher quantity of medication (i.e. three-monthly drug dispense), compared to the previous monthly consultation and dispensing model. Experts were convinced that reducing unnecessary health facility visits, not only reduces the patient load at the overburdened health facilities, it also removes some patient barriers, and maximises the time health professionals can spend with patients that are in a more critical condition, who may require more clinical attention and additional treatments (e.g. CPT).

*It is not so easy for patients to make all these journeys [monthly health facility visits]. One of the problems is transport costs. Not all patients have a favourable income. And if he needs to work, he rather goes to work. …Three-monthly dispense includes ART and CTZ, as well. That way, patients manage better to follow their preventive therapy.” (TB Manager, PDH).*

#### Drug delivery strategies

Differentiated care enables stable patients to bypass routine consultations, laboratory tests and psychosocial support sessions for six months, and proceed directly to the pharmacy to receive three-monthly medication, reducing the number of times each patient approaches the pharmacy. The provision of CPT (and ART) during routine consultations was a second alternative delivery approach mentioned by the experts. Last, if unable to obtain tablet refills themselves, patients also have the option to send a representative to collect their medicine.

*“One of the strategies to overcome barriers to CPT is the provision of CTZ during the consultation. He needs to wait only once and gets the medicine.” (TB Manager, DDH); “The ones that cannot make it [to the health facility] can send someone [with their patient card] to collect the therapy.” (HIV Manager, PDH)*

### Health care provider factors

#### Stock of health professionals

One expert noted that some health professionals working at the health facility were contracted by NGO’s, adding to the number of health professionals working for the public health system. On the contrary, the expert also pointed out that district health facilities suffered from losing health professionals who frequently choose to work with NGO’s outside the public healthcare service context.

*“Now that our partner [NGO] is hiring some health professionals to support the health facilities, I hope that we manage to overcome long queues.” “But it will continue to be challenging. We really struggle with staff. Health facilities are complaining because practically every month in some district, someone is leaving to work with an NGO, leaving for another system.” (HIV Manager, PDH)*

#### Training & health providers’ acceptability to prescribe CPT

Several experts reported that training facilitated the implementation of CPT. When probing experts from which sources health professionals involved in HIV care obtained their knowledge on CPT, on-the-job training for new employees and health facility-based continuous training for all health professionals, were the types of formal training reported.

*“Training health professionals on clinical guidelines, how to use CPT, who receives and who doesn’t receive CTZ, helped to implement CPT.” (TB Program Director, MoH); “At some point, health facilities started to organise continuous training where they spoke more about preventive therapies. We [district team] provided topics to [higher level] professionals to present during each training session.” (TB Manager, DDH)*

Another type of training mentioned was aimed at addressing service delivery issues within small groups of health care providers who had difficulties to correctly prescribe an intervention according to the eligibility criteria.

*“We usually tell health facilities to send new people and those that have more difficulties to [health facility-based] ART committee meetings. Get the one to explain the criteria for prescribing CPT, who we identified with difficulties when reviewing patient files. Same as we did at school, when the teacher came and said: “Today, you will present!” He probably chose you because he knew you had difficulties with the topic. Not everyone fails to prescribe because of irresponsibility, sometimes people do not know.” (HIV Manager, DDH).*

Experts also reported a training emphasis on ensuring that health professionals provide proper information about the benefits of CPT to patients newly diagnosed with HIV, to correctly stage HIV, and to initiate CPT during that first contact, if necessary. The introduction of CPT meant that, for the first time, health care providers had proven a biomedical intervention to offer to their HIV/AIDS patients in the absence of ART. Overall, great acceptability among health professionals to prescribe CPT, has been reported.

“There was a change from a time when HIV killed, AIDS killed and we had nothing we could give to the patient.” …"[With the introduction of CPT], providers were told never to miss the opportunity to offer CPT early during the first consultation. That was one of the strategies I found here at the district.” (HIV Manager, DDH) “Even if I am not sure how to medicate this [HIV], … it could be complicated, but we have CTZ at the pharmacy…” (HIV Manager, DDH)

### Patients’ perspectives

#### Patients’ strong preference for CTZ

Interviewees perceived that patients adequately adhered to CPT, and that they understood the importance of the preventive therapy. Patients frequently demand CTZ during clinical consultations, overall showing strong preference for the antibiotic, experts noted.

*“When you provide them with the medication [CTZ] and explain how they have to take it, they will adhere.” (HIV Manager, DDH); *

*“I find it hard to believe that there are patients that don’t know the importance. They are asking for it all the time …because they have been counselled and encouraged to take CTZ for improving their wellbeing. So patients are demanding CTZ: “I need these tablets.” (TB Manager, DDH);*

*Even if they don’t fulfil criteria, they believe that CPT somehow treats all diseases. When he arrives [to the health facility] with a cough, he believes CTZ will cure him. Patients like CTZ, they adore CTZ. I believe they like CTZ more than proper ART, they like CTZ a lot. (HIV Program Director, MoH);*

*“Often patients that did not receive CTZ, they came to your consultation asking… “Why didn’t I get?” Or some patients, after six months, their CD4 count was above 350 [cell/μL], and we suspended CPT. He looks at you and says: “Without this medicine, I will have problems!”(HIV Manager, DDH);*

*“Sometimes we speak to patients when they meet in their [patient-organised) adherence group. We sit down and discuss, and they complain: “We used to take this medicine [CTZ] before they [health providers] gave us ART, but now they don’t give it [CTZ] to us anymore, even though without it we don’t feel well.” (HIV Manager, PDH)*

### Focus group discussion

After interview content had been shared with each interviewee via email, and written authorisation to publish the content had been obtained, a focus group discussion (FGD) was organised (by PM, EM, MS), and held at the medical faculty of the Eduardo Mondlane University in April 2019. Focus group participants included three of the governmental stakeholders interviewed (one TB expert (PDH), two HIV experts (MoH, PDH)) and three additional stakeholders involved in policy implementation (the district Director, provincial Chief medical officer, and one HIV physician). Study findings were presented by EM and PM and discussed with the six focus group participants. The discussion was audio-recorded, transcribed, and analysed using the same approach described for interviews. The theme ‘patients’ strong preference for CTZ’ became the topic participants got most engaged in. Although focus group participants repeatedly emphasised how much patients like CTZ, they were struggling to explain the reason behind this phenomenon. No additional themes emerged from the discussion. Therefore, no repeat interviews had to be carried out.

## Discussion

**Fig. 1:**
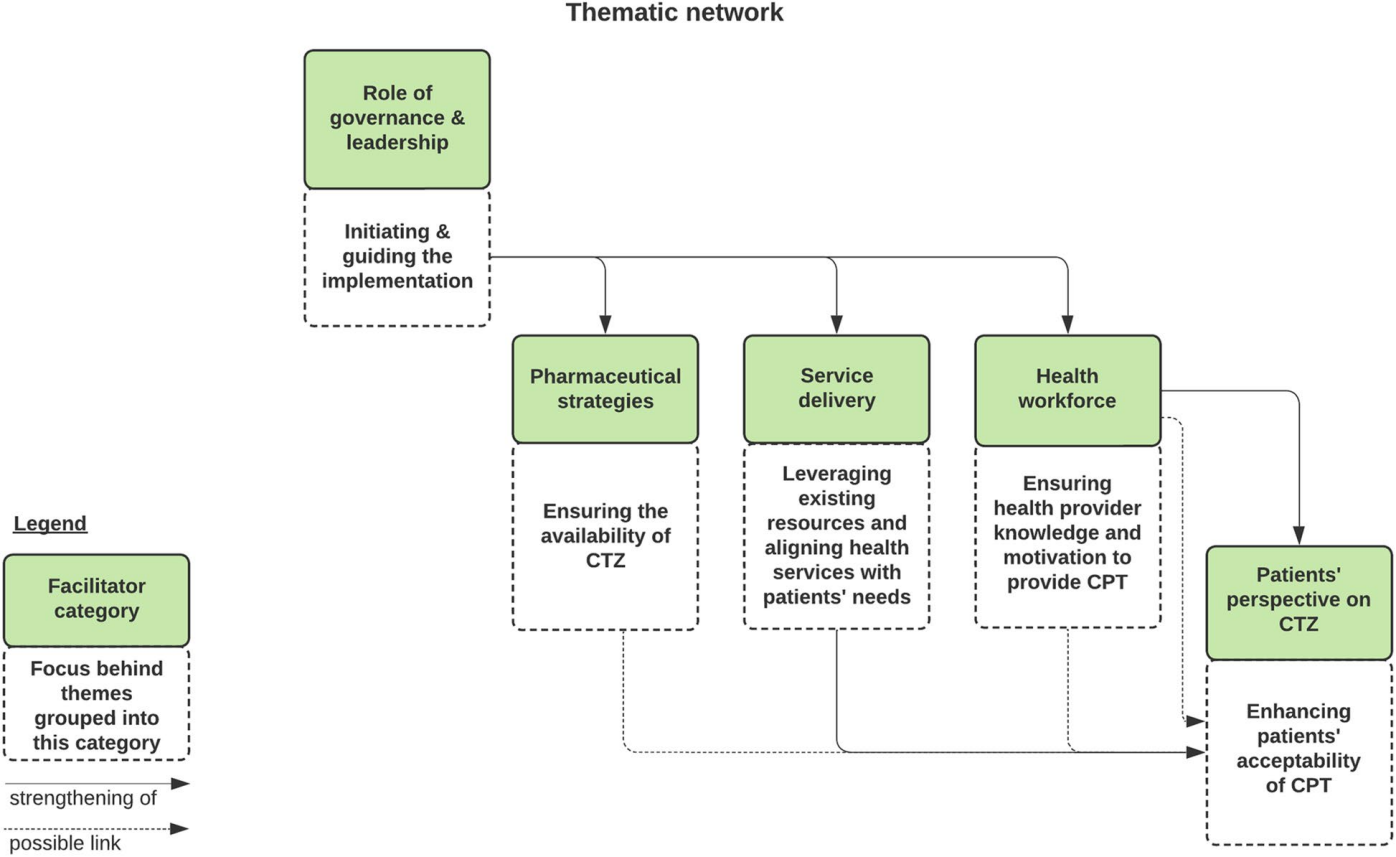
Thematic network analysis

Our paper is the first to our knowledge that studied facilitators for the implementation of CPT in a high HIV prevalence country that has reported remarkable progress implementing the preventive therapy. Thematic analysis of the content of interviews with nine governmental stakeholders revealed fifteen factors for the successful implementation of CPT in the Province of Maputo. Five clusters of themes (facilitator categories) resulted from thematic network analysis summarising the context-specific findings of our study (Fig. 1).

Key strategies included in each facilitator category are summarised in Additional file 3.

### Which factors and circumstances may have triggered initial policy development?

To begin with, our study suggests that good governance and leadership played a crucial role at the start of implementation (i.e. when defining the implementation strategy, providing official documents), and throughout the implementation process (i.e. ensuring continuous training and implementation progress). However, the decision to translate a global recommendation into national policy preceded these anticipated activities. In Mozambique, key actors within the government gave high priority to CPT; the fact that an intervention for PLHIV was urgently needed and the availability of funds for CPT appear to have triggered initial policy development. In line with the findings of Hutchinson et al. [11], who highlighted the importance of key actors (so-called “policy entrepreneurs”) in supporting CPT policy, our interviews indicate that key actors within the HIV Program and CMAM helped to get CPT on the policy agenda. Besides preventing opportunistic infections among PLHIV, governmental experts emphasized the perceived importance of CPT for reducing malaria infection in a country where malaria is transmitted perennially. However, the urgent need for an intervention is an additional explanation for the rapid policy translation. At the time, no treatment for HIV had been publicly available in Mozambique. During the pre-ART era, being diagnosed with HIV posed a real threat to people’s survival. In the year 2000, across the country, tens of thousands of people were severely immunocompromised and an estimated 45,000 died from AIDS [36]. “People died of AIDS, and health professionals had nothing they could give [to their patients with HIV/AIDS].”, one expert revealed. So we believe that a combination of misery and faith in an urgently needed intervention may have contributed to the rapid policy uptake and the priority given to implementing CPT. Finally, early in the policy development process, there has been explicit donor funding to implement CPT in Mozambique. The Mozambican government has ensured that there are sufficient funds to purchase CTZ, irrespective of the share of funding provided by donors, ever since. Similarly, others have reported that in Uganda, the availability of donor funding has facilitated policy development, while in Zambia, concerns about the economic feasibility of implementation have hindered policy uptake [10]. Contrary to the governments of other African countries, which delayed national policy development because they were not convinced that the available evidence justified policy implementation [11], the Mozambican government had promptly introduced its’ first policy on CPT in 2001 [37], suggesting that limited evidence was not a barrier to the initial policy decision in our study setting.

### Why has drug availability been better for CTZ than for other medicines?

Ensuring the availability of CTZ is crucial for implementing CPT successfully and sustainably. Pharmaceutical experts independently stated that overall, drug availability has been better for CTZ than for other medicines, which has led us to explore reasons for this favourable position. Interviewees reported (i) measures to ensure uninterrupted drug supply and (ii) measures to address issues concerning drug demand.

First, one major progress on the supply-side was that with the recent establishment of British pharmaceutical manufacturer STRIDES in Mozambique, the largest proportion of CTZ consumed in the Province of Maputo has been locally produced. National production of CTZ meant less dependence on foreign imports and more flexibility to cope with unexpected demand. Although local production of CTZ enabled CMAM to improve procurement performance (reduce costs, warehouse space and the year-long lead time for the procurement of kits from overseas), poor delivery performance at the end of the distribution pipeline made it impossible to remove CTZ from kits which arrived to public health facilities (PHFs) “on the fast lane”. Accordingly, kits continued to take an integral part of Mozambique’s pharmaceutical strategy, adding sturdiness to the supply chain. Pre-packed kits have been used around the world, since the early 1980’s to provide a selection of the most commonly needed basic medicines and supplies, either in emergency relief situations or for the routine supply of essential medicines to hard-to-reach health facilities. In contrast to the existing view that kits are a temporary measure to be replaced by a demand-oriented requisition system [38], in Mozambique, kits are delivered to all primary care PHFs, irrespective of their remoteness. For some PHFs that did not fulfil the minimum criteria for drug requisition (i.e. health centres/ health posts), kits remained the only option to obtain CTZ (and other essential medicines). For others kits served as a contingency plan that helped to overcome major logistic problems. Besides the logistic benefit of kits in Mozambique, which has also been shown by others [39], we argue that the “push-based supply” of medicines that is characteristic for kits, added robustness to the supply chain. Apparently, it was because health facilities always received kits in advance that ‘via clássica’ supply delays at least with regard to CTZ were not noticeable. The automated supply of kits ensured that basic stock of CTZ arrived at the health facilities (push strategy) even if the complementary requisition process (pull strategy) was delayed or has failed.

Second, among the strategic and operational measures put in place to reduce risks on the demand-side, the district management team played an important role. With the implementation of the requisition system, counting of drug inventory and estimating the medicine demand offered new challenges to health facility personnel. In line with previous findings [21], our interviews suggested that if orders were not placed in time, or if quantities were requested inadequately, health facilities would continue to face a mismatch of medicines available, or may even receive no supply at all. However, in our study setting, the district level manager took accountability, empowered facility personnel to correctly quantify and order medicines and organised stock transfers through a step-wise stock-seeking approach whenever facilities were facing a low-run on stock. Overall, the decentralisation of decision space and responsibility to a strong district management team that overlooks staff capacity and takes the final decision about drug re-allocation between health facilities or re-ordering from the nearest warehouse appeared to be a well-functioning system. Similarly, others have highlighted that decentralising logistic functions that require more knowledge about local conditions than central level experts generally have, can make the supply chain more effective [40].

### How has the delivery of CPT been aligned to address health system constraints and patient’s needs?

To set the scene, in the Province of Maputo, CPT and HIV care in general, are largely provided at the understaffed primary care PHFs. Managing the ever increasing number of patients requiring life-long HIV care with very few staff is far from perfect and certainly a difficult starting point for providing optimal health services and ensuring patients’ needs are met. However, successful and sustainable roll-out of CPT has been reported in our study setting, despite this challenge. The fact that prescription of CPT was never limited to medical doctors would have facilitated rapid scale-up since the beginning of implementation in 2001. Likewise, in Uganda [20] and South Africa [14] mid-level providers (clinical officers, nurses and midwifes) and lay counsellors have been entrusted with the task to deliver CPT. However, both studies also highlighted challenges associated with task shifting [14, 20], some of which can be addressed with clear policies, training [14] and on-site One-on-One mentorship [20]. While initial training and refresher training is crucial for the implementation of any intervention, in our study setting, district level program managers interactively re-emphasised eligibility criteria and the importance of CPT during routine ART committee meetings and supervisory meetings. The important role of management in the successful implementation of health interventions is often forgotten. However, the facilitators identified in this study indicate that top-down management commitment and support helped to ensure consistent policy implementation.

In more recent years, organisational changes at the health facility level seem to have improved service delivery and appear to have led to some urgently needed efficiency gains. According to the interviewees, the integration of care (i.e. prescription of CPT at several health facility sites), the introduction of differentiated care (i.e. 6-monthly consultation; 3-monthly drug dispense for stable HIV patients) and newer drug delivery strategies (i.e. direct pharmacy attendance, consultation-based CPT delivery and proxy attendance) have reduced long queues at the health facilities, and saved patients’ time and transport money. Considering the poor retention in care of PLHIV in the Province of Maputo (twelve months retention among PLHIV who newly initiated ART was 75% in 2018) [41], adjusting service delivery to align with patients’ needs appeared particularly important in our study setting. Our previously published review found that patients’ preference for traditional medicine can impede their acceptability of preventive therapies [12]. Therefore, the district level collaboration with traditional healers, mentioned by one interviewee, may be worth further investigating. Overall, organisational changes that have been described as beneficial for the implementation of CPT are likely to improve facility-based (HIV) care in general.

### What has led to patients’ strong preference for CPT?

Since our interview questions focused on policy updates and organisational changes, it was somewhat surprising, that patients’ positive influence on the success of the CPT Program was mentioned by governmental stakeholders across all Programs (HIV, TB, Pharmaceutical management) and all administrative levels. However, most of our interviewees had started their career working at PHFs. So sharing their experiences and observations from times when they worked at the frontline of delivery was natural. Although this phenomenon that patients like to take CTZ has been verified by focus group participants, it remains unclear why patients seem to find CTZ so appealing. Due to the great acceptability among health professionals to prescribe CPT, we speculate that health providers’ own conviction may have positively influenced patients’ attitudes towards the preventive therapy. In a Tanzanian study setting where the majority of health providers reported CPT was effective in preventing opportunistic infections, most parents/ guardians of children eligible for CPT also had a positive perception toward CTZ [16]. In other sub-Saharan study settings, improvements in quality of life and perceived health benefits after taking CPT, seemed to have encouraged patients attitude [22, 23]. However, one interviewee reported that patients give more importance to CTZ than to the actual medication for HIV (i.e. ART), suggesting that patients’ strong positive attitude toward one therapy could potentially threaten the uptake of another.

Although this study provides insightful findings, our results need to be interpreted with the following limitations on mind: First, we present exploratory findings that will require validation in future studies. However, even after demonstrating a significant positive effect of an implementation strategy (e.g. the decrease of CPT stock-outs at health facilities with district level supervision, compared to those without), transferability to other Provinces or other countries with a high burden of HIV, will still depend on local health system resources and context-specific barriers to implementation. Second, to study the case of the Province of Maputo, we aimed to explore the perspectives of stakeholders at all three different administrative levels (central, provincial and district level) and most relevant sectors (HIV, TB, pharmaceutical management). While central and provincial level experts were fully represented in this study, we only interviewed one team of district officers. Although the findings were often consistent across experts from the same sector, they may not accurately reflect potential district-level variation in experiences with the implementation of CPT among all eight districts located in the Province of Maputo. Third, since the study objective was presented to the interviewees at the beginning of the interview, social desirability bias may have influenced their responses. To support the accuracy of our findings and minimise confirmation bias potentially introduced during the process of data analysis and interpretation, the following validity strategies were incorporated into the study design: engagement of local native speaking researchers, cross checking of codes, enabling interviewees to review their transcripts, member checking, prolonged engagement, and focus group discussion with local stakeholders to promote critical reflection about the lessons learnt and to verify eventual saturation.

## Conclusions

Mozambique was among the first low-income countries in sub-Saharan Africa to implement CPT, and much priority is given at the central level to ensuring its coverage, until today. But what is there to be learnt from the Province of Maputo, over 20 years after the initial implementation of CPT? Among the five clusters of themes that emerged as facilitating factors for the implementation of CPT, three stood out. First, through recent years’ HIV service delivery modifications, feasibility of implementing CPT at the health facility level has improved. Since differentiated care and alternative drug delivery strategies had been rolled-out, unnecessary health facility visits and patient congestion at the health facilities have been reduced. As a result, health facilities seem to have been better equipped to manage the increasing number of patients entering HIV care, despite the health provider scarcity. Second, the present study casts a new light on reasons why shortages of CTZ were believed to be rather uncommon. Local production of the generic antibiotic and the combined push and pull distribution strategy appeared to improve the availability of CTZ, while drug accountability seemed to be triggered by decentralisation of responsibilities. However, shortages of other drugs (e.g. isoniazid) remain one of the Provinces’ unresolved issues, indicating that certain triggers, such as local production or multiple-channel drug distribution could be considered for other generic medicines, as well. Third, somewhat unexpected was the strong belief of our study participants that patients positively influenced the successful implementation of CPT. A better understanding of reasons for patients’ acceptability could inform policymakers and help shaping future implementation strategies.

### Supplementary Information

**Additional file 1.** Interview guide.

**Additional file 2.** COREQ checklist.

**Additional file 3.** Strategies for improving the implementation of CPT.

## Data Availability

The data underlying this article cannot be shared publicly for the privacy of individuals that participated in the study. The data will be shared on reasonable request to the corresponding author.

## Abbreviations

AIDS: Acquired immune deficiency syndrome
ART: Antiretroviral therapy
CMAM: Central de Medicamentos e Artigos Médicos (The central institution for medicines and medical Supply in Mozambique is a legal entity established by the Government which is responsible for procuring, storing, and distributing medicines and health products)
CPT: Cotrimoxazole preventive treatment
CTZ: Cotrimoxazole
DDH: District department of health
HIV: Human immunodeficiency virus
LMICs: Low and middle income countries
MCH: Maternal and child health
NGO: Non-governmental organization
PDH: Provincial department of health
PHFs: Public health facilities
PLHIV: People living with HIV
TB: Tuberculosis
WHO: World Health Organization

## Footnotes

1 The “Program for sexually transmitted infections, HIV/AIDS”, is referred to as “HIV Program”.

2 A technical working group is a multidisciplinary group of experts who work together on specific goals. Technical working group meetings allow stakeholders to discuss the state of research on a topic, the progress in policy development and implementation of a specific intervention.

3 Via clássica’ is the local term for drugs distributed through the pull-system. In other words, drugs that arrive at the health facility ‘via clássica’ have been ordered by authorized health facility personnel. Ordering drugs is reserved for larger health facilities that fulfil certain requirements, e.g. employment of at least one medical doctor).
