# Supplementary material for: Reasons for implementation success despite health system constraints: qualitative insights on ‘what worked’ for cotrimoxazole preventive therapy
Source: BMC Health Serv Res. 2024 Mar 27;24:379. doi: 10.1186/s12913-024-10631-x (PMC10967051; doi:10.1186/s12913-024-10631-x)
Supplement: Supplementary file 1 — Additional file 1. Interview guide. [file 12913_2024_10631_MOESM1_ESM.pdf]

Additional file 1. Interview guide

Name of the governmental expert: ______________________________________

Email: ____

Location of the interview: ____

Date: ____________________ From:______ Until: ______ Duration: ______min

Introduction & project presentation/ objectives
Script: Hello. My name is Pia Müller and this is my colleague Edna Mabasso, and we are pleased to meet you. Thank you for taking the time for this expert interview.

As mentioned [in my email / during our phone call] this research project is based on a collaboration between IHMT (Lisbon), UEM (Maputo), funded by FHB (Germany).

During our research trip to Mozambique in October 2017, we learnt that the implementation of CPT has been more successful here, in the province of Maputo than in other countries with a high burden of HIV and TB. That is why during this interview, we would like to learn from you which strategic decisions [on a national / provincial / district level] (e.g. financial, organizational, structural, political changes) have helped to overcome the barriers observed in other countries.

After the interview: Focus Group Discussion (FGD)
Script: At the end of this field research visit, we are going to organise a FGD, to which we would like to invite you and other experts who contributed to this research project. This FGD will take place on Tuesday, 30/04/2019, at the Medical Faculty of the Eduardo Mondlane University (UEM). During this meeting, we will share the preliminary results of the study and at that time we will encourage everyone to share their views and discuss the results. Irrespective of your availability to participate, all findings resulting from our interview will be presented to you before the FGD and your consent will be obtained via email prior to publication.

[Questions to be asked before obtaining written consent to participate on the interview:]

Script:

- Do you have any questions?
- Do you still agree to give us the interview?

[Obtaining written consent]

Script:

I would like to record the interview because it is easier to follow your story and analyse interview data afterwards. However, if you prefer, I will not record.

- Do I have your permission to record our interview?
- Are you ready to start the interview?

Interview questions for HIV/ TB experts:

1. How was it possible in the Province of Maputo to successfully implement CPT? Which strategies have helped to overcome previously existing barriers to CPT? What has been done here that has not been done elsewhere before?

2. Lately, which components of the [national/ provincial/ district] [TB / HIV strategy] have improved coverage of CPT?
   (Probe: anything particular about policies, guidelines, or protocols, technical assistance, feedback and refinement, organizational and financial changes, integrating TB and HIV services, increasing number of health facilities/ service providers, training: strengthening capabilities of individual providers and front-line workers, empowering communities/ households, supporting individual groups/ stakeholders, health system innovations, advocacy?)

3. From your perspective, why is the implementation of isoniazid more challenging than the implementation of cotrimoxazole? In your opinion from a [national / provincial / district] perspective, are there different challenges regarding the two preventive therapies?

4. Which is the biggest challenge in controlling HIV / TB in Mozambique?

5. How can the current CPT implementation strategy be improved? And IPT?

Questions for pharmaceutical experts:

1. How has Supply Management been improved in the last years to successfully deliver CPT and IPT to the health facilities? What has been done here that other countries can learn from? Which strategies have helped to overcome previously existing barriers to CPT? What has been done here that has not been done elsewhere before?

2. Lately, which components of the [national/ provincial/ district] drug supply strategy have improved the availability of Cotrimoxazole?
   (Probe: anything different in pharmaceutical planning, budget and prizing, procurement, storage, inventory management, distribution and use?)

3. From your perspective, why is the implementation of isoniazid more challenging than the implementation of cotrimoxazole? In your opinion from a [national / provincial / district] perspective, are there different challenges regarding the two preventive therapies?

4. Was an essential medicines list defined? If yes; does it include CPT and IPT?

5. How is (CPT and IPT) drug demand forecasted? (Probe: What is the demand calculation based on?)

6. Is there any local production of CPT or IPT? (Probe: Is local production an option?)

7. How is negotiation and management of pharmaceutical prizes organized? (e.g. annually, negotiated by the Ministry or including global drug facility)

8. What needs to be done in the future in your area of responsibility at the [national, provincial / district] level to overcome still existing barriers to CPT? and IPT?
