# Supplementary material for: Reasons for implementation success despite health system constraints: qualitative insights on ‘what worked’ for cotrimoxazole preventive therapy
Source: BMC Health Serv Res. 2024 Mar 27;24:379. doi: 10.1186/s12913-024-10631-x (PMC10967051; doi:10.1186/s12913-024-10631-x)
Supplement: Supplementary file 3 — Additional file 3. Strategies for improving the implementation of CPT. [file 12913_2024_10631_MOESM3_ESM.pdf]

Additional file 3. *Strategies for improving the implementation of CPT*

*for PLHIV, based on this study's findings

**How to initiate and guide the implementation?**	• Interdisciplinary elaboration of documents and implementation strategies<br>• Ensure health professionals have access to clearly written guidelines about the use of CPT (eligibility criteria, potential side effects, contraindications, when to start & stop CPT)<br>• Clear protocols for all levels and people responsible for supply chain operations<br>• Implement routine procedures that trigger continuous improvement (monitoring & evaluation activities, supportive supervision, routine health facility meetings to discuss service provision/ delivery issues)
**How to improve availability of CPT?**	• Prioritisation of CTZ (Essential medicines list, HIV program priority)<br>• Strategic focus on ensuring funding and the supply chain of CTZ<br>• Routine provision of pharmaceutical training for health facility personnel (requisition procedures, program priorities)<br>• Consider the supply of CTZ through two distinct supply channels (push and pull supply chain strategy)<br>• Include an appropriate quantity of buffer stuck<br>• Consider outsourcing troublesome steps of the supply chain to private companies<br>• Consider the possibility of local production of pharmaceuticals<br>• Consider decentralising activities related to the coordination of pharmaceuticals closer to the health facility (e.g. to district teams)
**Which health service- and drug delivery strategies to consider?**	• Consider extending health facility and or pharmacy opening hours<br>• Raising health literacy among patients and their community (increasing knowledge about CPT, reducing HIV related stigma)<br>• Consider integrating HIV services into primary care sub-specialities (paediatric, maternal and child health, chronic care, TB services)<br>• Consider collaborating with practitioners of traditional healing (TH) approaches (if TH is a common entry point into the health care system)<br>• Consider differentiated care models (reducing health facility visits among clinically stable patients)<br>• Consider alternative delivery approaches for CPT (e.g. community based provision to an adherence group representative or patient representative)
**How to ensure that CPT is prescribed/ delivered by health care providers?**	• Increasing the number of health professionals working within the NHS<br>• Routine training on CPT and mentoring for health professionals with difficulties regarding CPT prescription practices<br>• Ensuring health professionals' motivation and willingness to prescribe/ deliver CPT (attitude)
**How to increase patients**	• Increasing convenience (more timely access to HIV services, reliable access to CTZ)

**acceptability of CPT?**	• Allow patients to make informed decisions (increasing patients' self-motivation through key messages about the desired effects)
